# Editorial: Advancements in head and neck tumor treatment strategy driven by the perspective of precision oncology

**DOI:** 10.3389/fgene.2026.1886765

**Published:** 2026-06-09

**Authors:** Lingzi Liao, Qiushi Feng, Shang Xie

**Affiliations:** Department of Oral and Maxillofacial Surgery, Peking University School and Hospital of Stomatology and National Center for Stomatology and National Clinical Research Center for Oral Diseases and National Engineering Research Center of Oral Biomaterials and Digital Medical Devices, Beijing, China

**Keywords:** head and neck tumor, precision oncology, personalized medicine, tumor heterogeneity, cancer management

## 1 Introduction

Head and neck cancers impose a substantial health, socioeconomic, and psychological burden worldwide. Predominantly comprising head and neck squamous cell carcinoma (HNSCC), these malignancies account for approximately 890,000 new cases and 450,000 deaths annually, with nearly 65% of patients experiencing recurrence or metastasis (Chow, 2020). Current therapeutic strategies are primarily guided by clinical stage and pathological classification, which follow a comprehensive sequential treatment paradigm centered on surgical resection and/or definitive radiotherapy, adjuvanted by chemotherapy, and individualized use of targeted therapy, immunotherapy, rehabilitative supportive care and etc. (Dunn et al., 2026). However, pronounced intra-tumoral, inter-tumoral, and inter-patient heterogeneity, together with subclonal evolution, causes unpredictable variability in therapy responses across patients and tumor cell populations (Van den Bossche et al., 2022). Precision oncology is driving a major shift in cancer management: from empirical treatment regimens to individualized interventions guided by patient-specific molecular features, immune landscapes, and metabolic vulnerabilities (Okuyama et al., 2026). In this Research Topic, we present a Research Topic of studies that collectively advance precision oncology in head and neck cancers by integrating genomic, proteomic, and immunological approaches.

## 2 Anchoring the dynamics in tumor microenvironment

A key challenge in HNSCC treatment is the dynamic evolution of the tumor microenvironment (TME) and its role in therapeutic resistance. Precision risk stratification therefore requires biomarkers that capture both local TME features and systemic host responses. Within this context, Liu et al. established hyaluronan-mediated motility receptor (HMMR) as an independent prognostic biomarker in oral squamous cell carcinoma (OSCC), with elevated expression associated with advanced T/N stage, higher pathological grade, and adverse survival outcomes. Mechanistically, HMMR may drive OSCC progression by orchestrating Th2/T helper cell infiltration, tumor proliferation, hypoxic responses, DNA damage repair, and G2/M checkpoint regulation, with its interaction network encompassing key cell-cycle regulators such as CDK1, CCNB1, and AURKA. Collectively, these findings position HMMR as a promising candidate for OSCC risk stratification and precision therapeutic development. For systemic homeostasis, a blood-biochemistry analysis, directed by Cheng et al., demonstrated that inflammation-related indices, particularly Advanced Lung Cancer Inflammation Index (ALI) and Prognostic Nutritional Index (PNI), are closely associated with postoperative survival in OSCC. Notably, these indices effectively predicted short-term prognosis in male patients under 60 years of age, providing practical evidence for postoperative risk stratification, prognostic assessment, and individualized surveillance management in OSCC. Furthermore, An et al. introduced Repair Gene Promoter Methylation Burden (RPMB) as a novel, practical predictor for disease-free survival following adjuvant concurrent radio-chemotherapy (ARCT) in HNSCC, empowering clinicians to customize follow-up strategies for high-risk cohorts. Together, these studies expand HNSCC biomarker frameworks for precision risk stratification, supporting individualized prognosis, adjuvant therapy selection, and surveillance.

## 3 Multi-omics mapping for actionable target discovery

Building upon the prognostic frameworks established by clinical and microenvironmental biomarkers, precision oncology is fundamentally underpinned by high-resolution molecular mapping to unearth the underlying drivers of these risks. While comprehensive genomic databases exist, understanding regional and demographic mutational diversity is crucial for equitable precision medicine. Addressing this, Kunhabdulla et al. presented a specific genomic landscape analysis of OSCC from the southwest coast of Karnataka. By FFPE-based next-generation sequencing for paired tumor/mucosa samples from 21 cases, their work revealed region-specific mutational profiles, including shared mutations in genes such as ABCB1, CD44, IL6, PADI2, and VKORC1, tumor-exclusive TLR1 mutation, which provide a vital population-centered supplement to global genomic landscapes. Moving from the genome to actionable proteomic targets, Zhuang et al. employed integrated cytomembrane proteomics to identify EpCAM and MGST1 as promising therapeutic targets in metastatic laryngeal carcinoma. This bridging of membrane proteome exploration with translational target discovery lays the groundwork for the development of next-generation targeted interventions, such as antibody-drug conjugates, and investigation of their mechanisms such as Wnt/β-catenin–PI3K interruption.

## 4 Innovative intervention modalities and translational platforms

However, the identification of multi-omics targets is merely the prelude; the subsequent clinical imperative is to translate these molecular insights into effective treatments. Tailoring precise therapeutic interventions therefore remains the core principle and ultimate goal of precision oncology. Huo et al. showcased the potential of gene therapy by demonstrating that NK4 gene overexpression significantly attenuates the migratory activity of laryngeal squamous cell carcinoma cells, implicating the precise blockade of the HGF/Met signaling axis. However, translating these molecular interventions requires robust preclinical models that faithfully recapitulate patient-specific disease biology. Establishing a crucial bridge to clinical application, Chen et al. developed a groundbreaking platform utilizing HNSCC patient-derived organoids for adoptive cell therapy. This system successfully generated tumor-specific CD8^+^ T cells from peripheral blood lymphocytes (PBL), exemplifying functional precision medicine and enabling high-throughput screening to optimize personalized immunotherapy regimens prior to clinical administration. Taken together, these three dimensions, including microenvironmental risk stratification (diagnosis), omics-driven target discovery, and platform-based intervention strategies, form a cohesive, step-wise pipeline that propels head and neck precision oncology from theoretical concepts to therapeutic realities.

## 5 Future perspectives and conclusion

**FIGURE 1:**
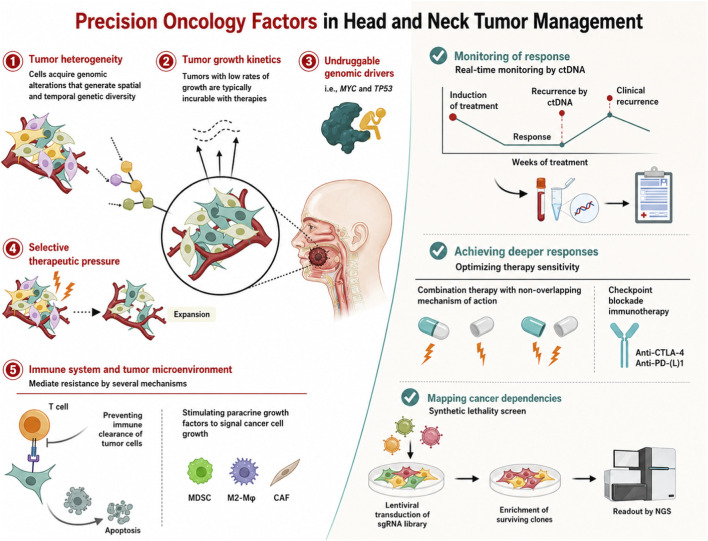
Precision oncology enhances the comprehensive management of head and neck tumor.

As the contributions to this Research Topic collectively underscore, the future development of precision oncology in head and neck cancer must move beyond conventional bulk-tumor profiling (Figure 1). However, precision oncology is not yet a flawless therapeutic paradigm. Constrained by tumor heterogeneity, the complexity of multi-omics interpretation, the cost of technical platforms, and barriers to clinical translation, many individualized strategies remain difficult to implement at scale in real-world practice (Polverini et al., 2018). Therefore, at the current stage, a more pragmatic trajectory for precision oncology may not be to design entirely new therapies for each patient from scratch, but rather to adapt adjustable components in the established systems of screening, diagnosis, treatment, surveillance, and prevention according to individual molecular features, population-specific backgrounds, and regional disease patterns. Although this approach may be less individualized than fully customized therapy, it offers greater clinical accessibility, scalability, and translational feasibility, better aligning precision medicine with routine clinical practice.

Looking ahead, addressing therapeutic resistance driven by cancer stem cells and tumor plasticity will require integrating higher-resolution multi-omics, such as single-cell RNA sequencing (scRNA-seq) and spatial transcriptomics, to decipher the precise developmental trajectories of malignant sub-clones. Furthermore, translating these insights into the clinic will increasingly rely on cross-disciplinary medical-engineering strategies. The integration of advanced biomaterials, functional nano-delivery systems, and innovative concepts like *in vivo* reprogramming holds immense promise for remodeling the immunosuppressive HNSCC microenvironment and precisely targeting cancer stem cell niches. By forging a closed-loop ecosystem from multi-omics discovery to organoid-based validation, and finally to bioengineered targeted delivery, we are steadily advancing toward the ultimate goal of curative, individualized care for patients with head and neck malignancies.

## References

[B1] Chow L. Q. M. (2020). Head and neck cancer. N. Engl. J. Med. 382, 60–72. 10.1056/NEJMra1715715 31893516

[B2] Dunn L. A. Ho A. L. Pfister D. G. (2026). Head and neck cancer: a review. Jama 335, 531–541. 10.1001/jama.2025.21733 41396597

[B3] Okuyama K. Naruse T. Matsushita Y. Fujimoto J. Yanamoto S. Lei Y. L. (2026). Precision immunotherapy for head and neck cancer: therapeutic combinations, biomarker strategies, and translational challenges. Mol. Cancer 25, 120. 10.1186/s12943-026-02609-6 41851770 PMC13130509

[B4] Polverini P. J. D'silva N. J. Lei Y. L. (2018). Precision therapy of head and neck squamous cell carcinoma. J. Dent. Res. 97, 614–621. 10.1177/0022034518769645 29649374 PMC5960884

[B5] Van Den Bossche V. Zaryouh H. Vara-Messler M. Vignau J. Machiels J. P. Wouters A. (2022). Microenvironment-driven intratumoral heterogeneity in head and neck cancers: clinical challenges and opportunities for precision medicine. Drug Resist Updat 60, 100806. 10.1016/j.drup.2022.100806 35121337

